# An Efficient Plaintext-Related Chaotic Image Encryption Scheme Based on Compressive Sensing

**DOI:** 10.3390/s21030758

**Published:** 2021-01-23

**Authors:** Zhen Li, Changgen Peng, Weijie Tan, Liangrong Li

**Affiliations:** 1 College of Computer Science and Technology, State Key Laboratory of Public Big Data, Guizhou University, Guiyang 550025, China; zli6@gzu.edu.cn (Z.L.); wjtan@gzu.edu.cn (W.T.); 2 College of Big Data and Information Engineering, Guizhou University, Guiyang 550025, China; lrli@gzu.edu.cn

**Keywords:** image encryption, compressive sensing, plaintext related, chaotic system

## Abstract

With the development of mobile communication network, especially 5G today and 6G in the future, the security and privacy of digital images are important in network applications. Meanwhile, high resolution images will take up a lot of bandwidth and storage space in the cloud applications. Facing the demands, an efficient and secure plaintext-related chaotic image encryption scheme is proposed based on compressive sensing for achieving the compression and encryption simultaneously. In the proposed scheme, the internal keys for controlling the whole process of compression and encryption is first generated by plain image and initial key. Subsequently, discrete wavelets transform is used in order to convert the plain image to the coefficient matrix. After that, the permutation processing, which is controlled by the two-dimensional Sine improved Logistic iterative chaotic map (2D-SLIM), was done on the coefficient matrix in order to make the matrix energy dispersive. Furthermore, a plaintext related compressive sensing has been done utilizing a measurement matrix generated by 2D-SLIM. In order to make the cipher image lower correlation and distribute uniform, measurement results quantified the 0∼255 and the permutation and diffusion operation is done under the controlling by two-dimensional Logistic-Sine-coupling map (2D-LSCM). Finally, some common compression and security performance analysis methods are used to test our scheme. The test and comparison results shown in our proposed scheme have both excellent security and compression performance when compared with other recent works, thus ensuring the digital image application in the network.

## 1. Introduction

Nowadays, digital images are becoming one of the most important data formats in our daily life. The risk of information leakage is inevitable when we share photos with others on the social network platform. Therefore, the security of digital images attracts a great of scholars’ attention.

The image encryption is an important method in image security. Many text structure encryption schemes, such as advanced encryption standard (AES), data encryption standard (DES), etc., have poor performance on image encryption. These schemes cannot break the correlation among adjacent pixels that may leak some geometric distribution of plain image. Image data are different from text data, which have some specific features, so the image encryption scheme must be designed according to these characteristics. At the beginning, the researchers used some special transformation matrixes, such as magic cube transformation, Arnold cat map, etc., in order to permutate the plain image without under security keys controlling. However, they are against Kerckhoffs’s principle, which requires the cryptosystem to be a white box, except for security keys.

Chaos theory as a cornerstone of nonlinear dynamic that is wildly used in many fields was first proposed by Lorenz [1]. The chaotic system has many good characteristics, such as randomness, ergodicity, sensitivity to initial values, and parameters [2], so it is suitable for the design of the cryptosystem. Matthews [3] first introduced a chaotic system into designing image cryptosystem. After that, a variety of image encryption algorithms have been put forward in the sspatial domain. These image encryption methods can be roughly classified as: (1) image encryption that is based on transformation matrixes [4,5,6]. This kind of algorithm mainly used the transformation matrix like magic cube transformation and Arnold cat map to permutation, and then used the chaotic system for diffusion. (2) The image encryption scheme that is based on deoxyribonucleic acid (DNA) encoding and chaotic system [7,8,9]. In this kind of scheme, the authors usually used DNA rules to encode the plain-image and controlled permutation and diffusion process on encoded data by the chaotic system. (3) Image encryption that is based on chaotic S-box [10,11,12]. Researchers usually used chaotic systems to design an S-box and encrypted image data by the nonlinear component. (4) Other spatial domain image encryption schemes [13,14,15,16].

Generally, the online transmission of image data requires a larger bandwidth. Therefore, image compression is very important for network applications, which can improve the efficiency of image transmission. In general, image compression not only utilizes the correlation between adjacent pixels, but it also encodes the non-uniform distribution of image pixels. However, image encryption will totally break the correlation among adjacent pixels and make its distribution uniform. Thus, the cipher image is not suitable for image compression. Not only that, the loss of image compression can also make the image impossible to be decrypted. Therefore, the compression must be executed early or at the same time as encryption.

Compressed sensing (CS) is a kind of effective data compression technology [17] when the data satisfy sparsity in a certain domain. When compared with Nyquist theory, CS can recover the entire signal from a smaller number of measurements [18]. In CS theory, when the signal is sparse in a transformation domain, a measurement matrix can be used to project the signal randomly, and then the original signal can be reconstructed by convex optimization algorithm. Fortunately, the images are sparse in many transform domains and are well suited to apply to CS theory. Therefore, based on compressed sensing, how to perform the image encryption also is a topical issue. Chai et al. [19] proposed an image encryption scheme that is based on magnetic-controlled memristive chaotic system and compressive sensing. This scheme first transform image to discrete wavelet transform (DWT) domain. Subsequently, some permutations have been done with this coefficient matrix. Finally, the compressive sensing used a measurement matrix that was generated by a chaotic system. In this scheme, although the generation of measurement matrix is related to plain image information, the plain image sensitivity of the scheme is still not good enough. In addition, the uneven energy distribution of cipher images generated by the scheme may also cause the leakage of some plain image information. Zhu et al. [20] proposed an image encryption scheme, which uses the random Gaussian matrix generated by Chebyshev mapping to execute compressive sensing. Chai et al. [21] proposed a chaotic image encryption scheme, which uses elementary cellular automata and block compressive sensing. In this scheme, a plain image is transformed by DWT at first, then compressive processing under the measurement matrix generated by a parameter-varying chaotic system is done. The plain image sensitivity of this scheme is good, because all of the initial values of chaotic system are related to the plain image. However, the randomness of cipher image seems not good enough. Zhu et al. [22] proposed an image encryption scheme, which is based on nonuniform sampling by block CS. In this scheme, the discrete cosine transform (DCT) is used to generate the coefficients matrix, and then perform compressive sensing processing by two measurement matrices that are generated by the logistic map. Finally, undertake the diffusion and permutation under logistic map controlling. However, the whole diffusion process is related to the result that is calculated in previous pixels; thus, the robustness must not be as good as mentioned. Gong et al. [23] proposed an image compression and encryption algorithm. In this scheme, the plain image is first permuted by the Arnold transform to reduce the block effect in the compression process, and then the coefficient matrix is compressed and encrypted by CS, simultaneously. The keys in this scheme are generated by a plain image without any external keys; it means that each cipher image corresponds to a unique key, which is not conducive to key distribution management and batch image encryption. Kayalvizhi et al. [24] proposed an image encryption scheme, which is based on compressive sensing, fractional order hyper chaotic Chen system, and DNA operations. In this scheme, block compressive sensing is executed to the plain image, and then execute DNA encoding to the measurement matrix. After that, complete some diffusion operation in DNA sequences. The whole process is not related to the plain image and the sensitivity of plain image has weak resistance to differential attack. Moreover, DNA encoding and decoding may consume a large amount of computing time, which results in the low efficiency of this algorithm.

To conquer the drawback what mentioned above, a plaintext related image encryption scheme is given using compressive sensing and two hyper chaotic systems. The detailed contributions are as follows:

- In order to make the image cryptosystem more sensitive to the plain image, a plain image information-related method is proposed, which makes the plaintext information involved in the whole control process of compressive sensing and encryption, and make the image cryptosystem have excellent performance in resisting differential attack.
- The generation method of the measurement matrix for compression encryption is presented, which is based on a chaotic system and the information of plain images, and make the CS process fully related to the plain image. In other words, different plain images correspond to different measurement matrices. Additionally, a permute and diffuse operation is used for the measurement matrix, which makes the pixels of the cipher image present lower correlation and uniform distribution.
- The peak signal to noise ratio and structural similarity index measurement is used to evaluate compression performance, and many common security analyses methods are carried out, such as key space analysis, differential attack, statistical analysis, key sensitivity analysis, etc., in order to evaluate security performance.

This paper is organized, as follows: in Section 2, the preliminary for this paper is given, such as compressive sensing and chaotic system. In Section 3, an efficient image encryption scheme that is based on chaos and compressive sensing is introduced. In Section 4, some common compression analyses and security analyses of the proposed image cryptosystem are given. In Section 5, we conclude this paper.

## 2. Preliminary

### 2.1. Compressive Sensing

The aim of CS model [25] is to recover a sparse image signal $X\in R^{n\times n}$ from fewer measurements $Y\in R^{m\times n}$ is given by:

(1) Y=ΦX=ΦΨP

where $\Phi \in R^{m\times n}$ is a measurement sensing matrix whose distribution satisfies Gaussian distribution. Let A=ΦΨ, where the columns satisfy the linearly independent condition. When A satisfies a certain condition, i.e., Restricted Isometry Constant (RIC), the restricted isometry property(RIP), the CS theory shows that only a sufficiently sparse signal P can be recovered with a high probability exactly from Y. The linear measurement process is expressed as a regularized form, as

(2) $${\min∥vec\left(P\right)∥}_{0}\;s.t.\;{∥AP-Y∥}_{2}\leq \eta$$

where ${∥\cdot ∥}_{0}$ denotes the $l_{0}$ norm as a sparsity constraint and η is a constant. This form aims to find the most sparse solution that fits the observation model well. However, it is Non-deterministic Polynomial (NP)-hard problem to solve Equation (2). A convex relaxation method is to apply the $l_{1}$ norm of the $l_{0}$ norm, as follows:

(3) $${\min∥vec\left(P\right)∥}_{1}\;s.t.\;{∥AP-Y∥}_{2}\leq \eta$$

Theoretical analysis has shown that the $l_{1}$ norm can also approach the most sparse solution under some conditions [25,26]. Equation (3) can be solved by some optimization algorithms, such as the gradient descent method (GDM) [27] abd orthogonal matching pursuit (OMP) [28].

### 2.2. Chaotic System

The chaotic systems in our proposed scheme are used to control the permutation and diffusion process, and to generate a measure matrix of compressive sensing, which are the key points of encryption and compression performance. Therefore, our cryptosystem is required to choose hyper chaotic systems that have better chaotic characteristics.

The two-dimensional Sine improved Logistic iterative chaotic map (2D-SLIM) [29] is given by

(4) $$\left\{\begin{array}{c} x_{i+1}=\sin\left(by_{i}\right)\sin\left(50/x_{i}\right)\\ y_{i+1}=a\left(1-2x_{i+1}^{2}\right)\sin\left(50/y_{i}\right) \end{array}\right.$$

where *a* and *b* are the system parameters. When a∈(0,3] and b=2π or when a=1 and b∈[4,7], the system becomes hyper chaotic.

The two-dimensional Logistic-Sine-coupling map (2D-LSCM) [30] is given by

(5) $$\left\{\begin{array}{c} x_{i+1}=\sin\left(\pi \left(4\theta x_{i}\left(1-x_{i}\right)+\left(1-\theta \right)\sin\left(\pi y_{i}\right)\right)\right)\\ y_{i+1}=\sin\left(\pi \left(4\theta y_{i}\left(1-y_{i}\right)+\left(1-\theta \right)\sin\left(\pi x_{i+1}\right)\right)\right) \end{array}\right.$$

where θ is the control parameter. When θ∈(0,1), the system has hyper chaotic behavior.

**Remark** **1.** *In our proposed scheme, we used 2D-SLIM and 2D-LSCM, two discrete hyper chaotic systems, to control the encryption process. In fact, other discrete hyper chaotic systems can also be extended in our scheme, and the only difference in those selection is the size of key space. Furthermore, the reason why we select the two different hyper chaotic systems to control the encryption and compression process is that it can avoid, as much as possible, some unexpected situations occurring, such as dynamical degradation of chaotic systems [31,32], weak real keys, etc.*

## 3. Our Proposed Scheme

The image cryptosystem is proposed in this section. First, a plaintext related internal keys generation method is introduced in Section 3.1. Afterwards, we present the encryption scheme in Section 3.2. Finally, we propose the decryption scheme in Section 3.3.

### 3.1. Plaintext-Related Internal Keys Generation

In this subsection, we proposed a method for generating the internal keys that are related to plaintext. The internal keys are used to generate the initial values and parameters of hyper chaotic systems that are used to control all processes of encryption and decryption. Therefore, the plaintext-related internal key generation method can make our proposed image cryptosystem more plaintext sensitive to resisting differential attack. There are two parts in plaintext-related internal key generation: plaintext information extraction and internal keys generation.

Algorithm 1 shows the plain image information extraction algorithm.

The detailed description are as follows:

***Step 1:*** input plain image matrix ***P*** and initial key ***K*** into algorithm, and begin.

***Step 2:*** expand the plain image matrix ***P*** into a vector ***P(:)*** in rows, and then change this vector to a string ***SP***.

***Step 3:*** input string ***SP*** into hash function SHA256, and denote the hash value as ***HP***.

***Step 4:*** input initial key ***K*** into hash function SHA256, and denote the hash value as ***HK***.

***Step 5:*** put hash values ***HP*** and ***HK*** together and input them into hash function SHA256. The hash value is extracted plain image information ***EPI***.

***Step 6:*** output the extracted plain image information ***EPI***, and finished.

**Algorithm 1** Plain image information extraction**Input:** Plain image matrix ***P*** and initial key ***K*** **Output:** Extracted plain image information ***EPI***.
1:***String SP***←***P(:)***2:***HP***← SHA256(***SP***)3:***HK***← SHA256(***K***)4:***EPI***← SHA256(***[HP,HK]***)


Algorithm 2 shows the internal keys generation algorithm.
**Algorithm 2** Internal keys generation**Input:** Extracted plain image information ***EPI***, initial key ***K*** **Output:** Internal keys ***[K1,K2,K3,K4]***.
1:***HK***← SHA256(***K***)2:***INKEY***← SHA256(***[EPI,HK]***)3:I1←INKEY(1:64)4:I2←INKEY(65:128)5:I3←INKEY(129:192)6:I4←INKEY(193:256)7:***CN1***$\leftarrow mod(I1/10^{8},\;256)$***CN2***$\leftarrow mod(I2/10^{8},\;256)$8:***CN3***$\leftarrow mod(I3/10^{8},\;256)$***CN4***$\leftarrow mod(I4/10^{8},\;256)$9:K1←BitCyclicShift(INKEY,CN1)10:K2←BitCyclicShift(INKEY,CN2)11:K3←BitCyclicShift(INKEY,CN3)12:K4←BitCyclicShift(INKEY,CN4)

The detailed description are as follows:

***Step 1:*** input extracted plain image information ***EPI*** and initial key ***K***, and begin.

***Step 2:*** input initial key ***K*** into hash function SHA256, and denote the hash value as ***HK***.

***Step 3:*** put extracted plain image information ***EPI*** and hash value ***HK*** together and input them into hash function SHA256. Denote the hash value as ***INKEY***.

***Step 4:*** split ***INKEY*** into four parts, and everypart with 64 bits, denoted as ***I1***, ***I2***, ***I3*** and ***I4***.

***Step 5:*** calculate the control values as $\mathrm{CN}1=mod(I1/10^{8},\;256)$, ***CN2***$=mod(I2/10^{8},\;256)$, ***CN3***$=mod(I3/10^{8},\;256)$, and ***CN4***$=mod(I4/10^{8},\;256)$.

***Step 6:*** bit cyclic shift ***INKEYCN1*** bits to right direction, and generate 256 bits internal key ***K1***, after that, at same operation to bit cyclic shift ***INKEY*** under ***CN2***, ***CN3***, ***CN4*** control, and generate internal keys ***K2,K3,K4***.

***Step 7:*** output internal keys ***[K1,K2,K3,K4]***, and finished.

### 3.2. Encryption Scheme

**Figure 1:**
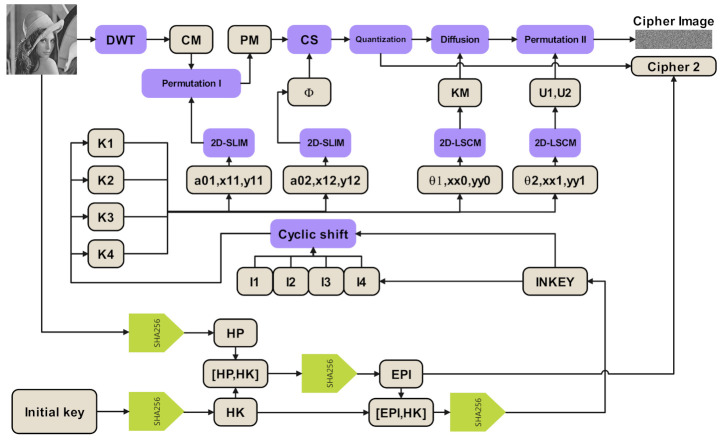
The block diagram of the encryption scheme.

In this subsection, we will introduce our proposed encryption scheme. The encryption scheme takes, as inputs, plain image ***P***, initial key ***K***, and compression ratio **CR**, and put outputs, such as cipher image and some additional ciphertext information. The compression ratio (CR) means the ratio of the number of pixels in the compressed image to that in the original image. Figure 1 shows the block diagram of the encryption scheme, and the detailed description are as follows:

***Step 1:*** input plain image ***P*** (N×N), initial key ***K*** and compression ratio **CR**, and the encryption process begins.

***Step 2:*** input plain image matrix ***P*** and initial key ***K*** into Algorithm 1 to get extracted plain image information ***EPI***. After that, input extracted plain image information ***EPI*** and initial key ***K*** into Algorithm 2 to generate internal keys ***[K1,K2,K3,K4]***.

***Step 3:*** input plain image ***P*** into discrete wavelet transform (DWT) to sparse representation, and we denote sparse coefficient matrix as ***CM***.

***Step 4:*** input coefficient matrix ***CM*** and internal key ***K1*** into Algorithm 3 to make plaintext energy evenly distributed.
**Algorithm 3** Permutation I algorithm**Input:** Coefficient matrix ***CM*** and internal key ***K1*** **Output:** Permutated coefficient matrix ***PM***.
1:I1←K1(1:64); I2←K1(65:128); I3←K1(129:192)2:$a01\leftarrow fix\left(mod\left(I1/10^{6},\;3\right)\right)+mod\left(I1/10^{14},\;1\right)$; b←2π3:$x11\leftarrow mod(I2/10^{14},\;1)$; $y11\leftarrow mod(I3/10^{14},\;1)$4:Put a01,b,x11,y11 into Equation (4) to generate a sequence S by iterating.5:$X\leftarrow mod(fix\left(\left(S+100\right)\times 10^{10}\right),N\times N)+1$6:Remove the repeated elements from ***X***, put the absent numbers at the end.7:Change ***CM*** to a vector ***CMA*** in rows.8:len←length(CMA)9:**for**i=1**to**fix(len/2)**do**10: CMA(X(i))↔CMA(X(len−i+1))
11:**end for**12:***PM***←reshape(CMA,N,N)


***Step 5:*** Calculate the threshold value ***TS*** by Algorithm 4.
**Algorithm 4** Calculate threshold algorithm**Input:** Coefficient matrix ***CM*** and compression ratio **CR** **Output:** Threshold value ***TS***.
1:Change ***CM*** to a vector ***CMA*** in rows.2:Arrange the vector ***CMA*** from the smallest to the largest3:len←length(CMA)4:***TS***←CMA(floor(len−N×N× CR /7))


***Step 6:*** if the element in the permutated coefficient matrix ***PM*** absolute value less than threshold ***TS***, then set this element to 0. The new generated matrix is denoted as ***PM2***.

***Step 7:*** input matrix ***PM2***, compression ratio **CR**, and internal key ***K2*** into Algorithm 5 to obtain the measurements ***CSM***.
**Algorithm 5** Compressive sensing algorithm**Input:** The matrix ***PM2***, compression ratio ***CR***, and internal key ***K2*** **Output:** The compressive sensing measurements ***CSM***.
1:I1←K2(1:64)2:I2←K2(65:128)3:I3←K2(129:192)4:$a02\leftarrow fix\left(mod\left(I1/10^{6},\;3\right)\right)+mod\left(I1/10^{14},\;1\right)$;5:b←2π6:$x12\leftarrow mod(I2/10^{14},\;1)$7:$y12\leftarrow mod(I3/10^{14},\;1)$8:M←fix(N×CR)9:Put a02,b,x12,y12 into Equation (4) to generate a sequence S by iterating M×N times.10:Φ0←reshape(S,M,N)11:$\Phi \leftarrow \sqrt{2/M}\cdot \Phi 0$, where $\sqrt{2/M}$ is used for normalization [33].12:CSM←PM2×Φ

***Step 8:*** quantize the compressive sensing measurements ***CSM*** to the range of [0,255] and generate quantized matrix ***QM*** by

(6) $$\mathrm{QM}=\;\mathrm{round}\;\left(255\times \left(\mathrm{CSM}-\mathrm{MAX}\right)/\left(\mathrm{MAX}-\mathrm{MIN}\right)\right)$$

where round(x) represents the nearest integer with *x*, and MIN and MAX are the minimum and maximum numbers of ***CSM***.

***Step 9:*** input quantized matrix ***QM*** and internal keys ***K3,K4*** into Algorithm 6 to do diffusion and permutation II.

***Step 10:*** output cipher image ***C1*** and additional cipher information C2=[EPI,MAX,MIN]. The encryption process is finished.
**Algorithm 6** Diffusion and permutation II algorithm**Input:**Quantized matrix ***QM*** and internal keys ***K3,K4*** **Output:** Cipher image ***C1***.
1:I1←K3(1:64); I2←K3(65:128); I3←K3(129:192); I4←K4(1:64); I5←K4(65:128); I6←K4(129:192).2:$\theta 1\leftarrow mod(I1/10^{14},\;1)$; $xx0\leftarrow mod(I2/10^{14},\;1)$; $yy0\leftarrow mod(I3/10^{14},\;1)$.3:$\theta 2\leftarrow mod(I4/10^{14},\;1)$; $xx1\leftarrow mod(I5/10^{14},\;1)$; $yy1\leftarrow mod(I6/10^{14},\;1)$.4:**if**0.33<θ1<0.66**then**5: θ1←mod(θ1+0.33,1)
6:**end if**7:**if**0.33<θ2<0.66**then**8: θ2←mod(θ2+0.33,1)
9:**end if**10:[M,N]←size(QM)11:Put θ1,xx0,yy0 into Equation (5) to generate a sequence S0 by iterating M×N times.12:$\mathrm{KM}\leftarrow floor(mod\left(S0\times 10^{13},256\right))$13:DM←QM⨁KM14:Put θ2,xx1,yy1 into Equation (5) to generate a sequence X1 and Y1 by iterating max(M,N) times.15:$U1\leftarrow floor(mod\left(X1\left(1:M\right)\times 10^{5},\left(M-1\right)\right))+1$16:$U2\leftarrow floor(mod\left(Y1\left(1:N\right)\times 10^{5},\left(N-1\right)\right))+1$17:**for**i=1**to***M***do**18: DM(i,:)←CircleShift(DM(i,:),U1(i))
19:**end for**20:**for**i=1**to***N***do**21: DM(:,i)←CircleShift(DM(:,i),U2(i))
22:**end for**23:C1←DM


### 3.3. Decryption Scheme

**Figure 2:**
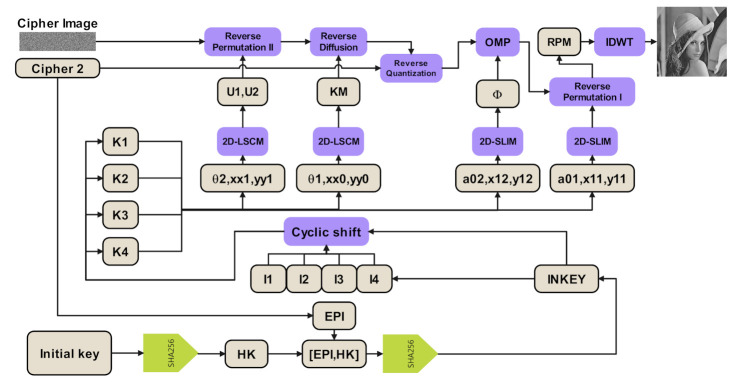
The block diagram of the decryption scheme.

The decryption process is the inverse process of encryption, and it takes input as cipher image ***C1***, additional cipher information ***C2***, and initial key ***K***, and put the output as recovering plain image. Figure 2 shows the block diagram of the decryption scheme, and the detailed description is as follows:

***Step 1:*** input cipher image ***C1***(M×N), additional cipher information ***C2***, and initial key ***K*** and the decryption process begins.

***Step 2:*** input ***EPI*** and initial key ***K*** into Algorithm 2 to generate internal keys ***[K1,K2,K3,K4]***.

***Step 3:*** input cipher image ***C1*** and internal keys ***K3,K4*** into Algorithm 7 to do reverse permutation II and reverse diffusion.
**Algorithm 7** Reverse permutation II and reverse diffusion algorithm**Input:** Cipher image ***C1*** and internal keys ***K3,K4*** **Output:** Reverse permutation and diffusion matrix ***RPDM***.
1:I1←K3(1:64); I2←K3(65:128); I3←K3(129:192); I4←K4(1:64); I5←K4(65:128); I6←K4(129:192).2:$\theta 1\leftarrow mod(I1/10^{14},\;1)$; $xx0\leftarrow mod(I2/10^{14},\;1)$; $yy0\leftarrow mod(I3/10^{14},\;1)$.3:$\theta 2\leftarrow mod(I4/10^{14},\;1)$; $xx1\leftarrow mod(I5/10^{14},\;1)$; $yy1\leftarrow mod(I6/10^{14},\;1)$.4:**if**0.33<θ1<0.66**then**5: θ1←mod(θ1+0.33,1)
6:**end if**7:**if**0.33<θ2<0.66**then**8: θ2←mod(θ2+0.33,1)
9:**end if**10:[M,N]←size(C1)11:Put θ2,xx1,yy1 into Equation (5) to generate a sequence X1 and Y1 by iterating max(M,N) times.12:$U1\leftarrow floor(mod\left(X1\left(1:M\right)\times 10^{5},\left(M-1\right)\right))+1$13:$U2\leftarrow floor(mod\left(Y1\left(1:N\right)\times 10^{5},\left(N-1\right)\right))+1$14:**for**i=1**to***N***do**15: C1(:,i)←CircleShift(C1(:,i),−U2(i))
16:**end for**17:**for**i=1**to***M***do**18: C1(i,:)←CircleShift(C1(i,:),−U1(i))
19:**end for**20:Put θ1,xx0,yy0 into Equation (5) to generate a sequence S0 by iterating M×N times.21:$\mathrm{KM}\leftarrow floor(mod\left(S0\times 10^{13},256\right))$22:RPDM←C1⨁KM


***Step 4:*** Do reverse quantization to the matrix ***RPDM***, and generate reverse quantized matrix ***RQM*** by

(7) $$\mathrm{RQM}=\frac{\mathrm{RPDM}\times (\mathrm{MAX}-\mathrm{MIN})}{255}+\mathrm{MIN}$$

***Step 5:*** Input matrix ***RQM*** and internal keys ***K2*** into Algorithm 8 to reconstruct matrix ***RCM***.

***Step 6:*** input reconstruct matrix ***RCM*** and internal key ***K1*** into Algorithm 9 to undertake reverse permutation I.

***Step 7:*** input matrix ***RPM*** into inverse discrete wavelet transform (IDWT) in order to recover plain image ***P***.

***Step 8:*** output recover plain image ***P*** and the decryption process is finished.
**Algorithm 8** Matrix reconstruction algorithm**Input:** Matrix ***RQM*** and internal keys ***K2*** **Output:** Reconstruct matrix ***RCM***.
1:I1←K2(1:64)2:I2←K2(65:128)3:I3←K2(129:192)4:$a02\leftarrow fix\left(mod\left(I1/10^{6},\;3\right)\right)+mod\left(I1/10^{14},\;1\right)$;  b←2π5:$x12\leftarrow mod(I2/10^{14},\;1)$6:$y12\leftarrow mod(I3/10^{14},\;1)$7:[M,N]←size(RQM)8:Put a02,b,x12,y12 into Equation (4) to generate a sequence S by iterating M×N times.9:Φ0←reshape(S,M,N)10:$\Phi \leftarrow \sqrt{2/M}\cdot \Phi 0$, where $\sqrt{2/M}$ is used for normalization.11:**for**i=1 to *N* **do**12:RCM(:,i)←OMP(RQM,Φ,N)13:**end for**
**Algorithm 9** Reverse permutation I algorithm**Input:** Reconstruct matrix ***RCM*** and internal key ***K1*** **Output:** Reverse permutated matrix ***RPM***.
1:I1←K1(1:64)2:I2←K1(65:128)3:I3←K1(129:192)4:$a01\leftarrow fix\left(mod\left(I1/10^{6},\;3\right)\right)+mod\left(I1/10^{14},\;1\right)$;5:b←2π6:$x11\leftarrow mod(I2/10^{14},\;1)$7:$y11\leftarrow mod(I3/10^{14},\;1)$8:Put a01,b,x11,y11 into Equation (4) to generate a sequence S by iterating.9:$X\leftarrow mod(fix\left(\left(S+100\right)\times 10^{10}\right),N\times N)+1$10:Remove the repeated elements from ***X***, put the absent numbers at the end.11:Change ***RCM*** to a vector ***RCMA*** in rows.12:len←length(RCMA)13:**for**i=1**to**fix(len/2)**do**14: RCMA(X(i))↔RCMA(X(len−i+1))
15:**end for**16:***RPM***←reshape(RCMA,N,N)


## 4. Simulation and Analysis

**Figure 3:**
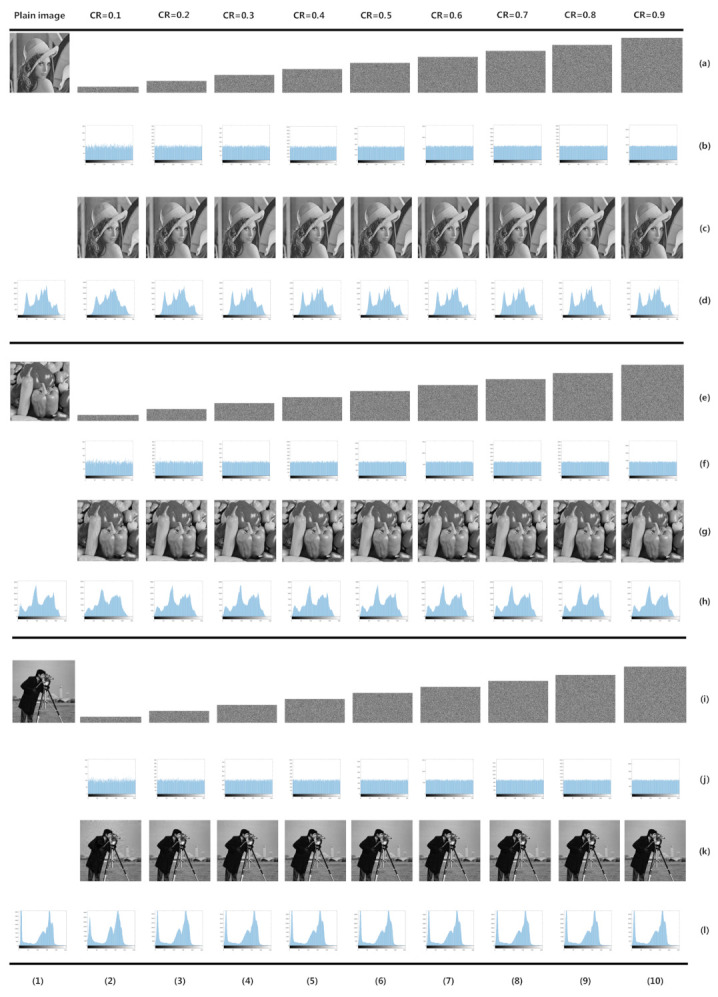
Encryption simulation. Rows (**a**–**d**) are the simulation results of image ’Lena’, Rows (**e**–**h**) are the simulation results of image ’Pepper’ and Rows (**i**–**l**) are the simulation results of image ’Cameraman’. Column (**1**) are plain images. Columns (**2**–**10**) are the simulation results of CR = 0.1, ⋯, CR = 0.9, respectively.

In this section, we will evaluate our proposed image cryptosystem. The simulations and performance evaluations are implemented in MATLAB R2016a. Our hardware environment for tests was a personal computer with Inter(R) Core i7-6700k CPU 4.00 GHz, 32 GB memory, and the operation system is Windows 7 home edition. For simulation and tests, the initial key is selected as ‘a2b235c5dd4345d2445e33e25ef255f524235ec’ in hexadecimal, and one of the parameters of 2D-SLIM, which is given in Equation (4), is set as b=2π. We first select 512×512 8-bit level gray images ’Lena’, ’Pepper’, and ’Cameraman’ for simulation, and encrypt them with CR = 0.1,0.2,⋯,0.9, respectively. Figure 3 shows the simulation result. In the following subsections, we first discussed the performance of compression, and then provided the common security analysis result of our proposed image cryptosystem. Finally, we compared our work with other recent works in order to make our proposed image cryptosystem more convincing.

### 4.1. Compression Analyses

#### 4.1.1. Peak Signal to Noise Ratio (PSNR)

**Figure 4:**
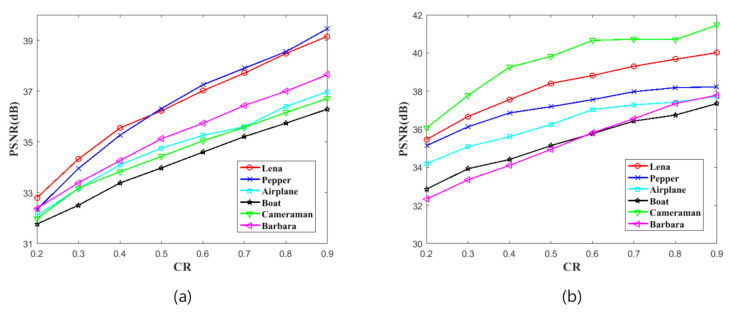
The Peak Signal to Noise Ratio (PSNR) test result. (**a**) shows the results of images with the size of 256×256. (**b**) shows the results of images with the size of 512×512.

For measuring the difference between the decrypted image and the original image to evaluate the recovery quality, we use the peak signal to noise ratio (PSNR) as a measurement for evaluation. PSNR is given by

(8) $$\mathrm{PSNR}=10\log_{10}\frac{255\times 255}{\frac{1}{N^{2}}\sum_{i=1}^{N}\sum_{j=1}^{N}{\left(I\left(i,j\right)-I^{\prime }\left(i,j\right)\right)}^{2}}$$

where *I* and $I^{\prime }$ are the decrypted image and original image, respectively. In this test, we first encrypt 256×256 and 512×512 plain images at different CRs, and then decrypt these cipher images to obtain recovery images. Finally, we calculate PSNR between plain images and recovery images. Table 1 and Figure 4 show the test result. According to the results, the PSNR values between plain images and recovery images are increasing with the growth of CRs, and the minimum of the PSNR is 31.7675 dB when the image is 256×256 and be encrypted in CR =0.2. Therefore, our proposed scheme has a very good compression recovery performance.

#### 4.1.2. Structural Similarity Index Measurement (SSIM)

**Figure 5:**
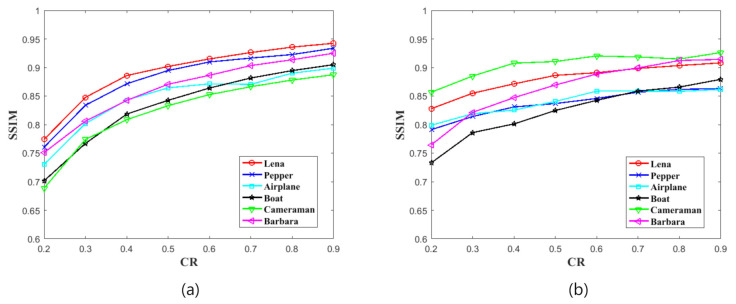
The Structural Similarity Index Measurement (SSIM) test result. (**a**) is shown the results of images with the size of 256×256. (**b**) is shown the results of images with the size of 512×512.

The structural similarity index measurement (SSIM) is another important indictor for evaluating the compression performance. The SSIM value can be calculated by

(9) $$\mathrm{SSIM}=\frac{\left(2\mu_{I}\mu_{I^{\prime }}+C_{1}\right)\left(2\sigma_{II^{\prime }}+C_{2}\right)}{\left(\mu_{I}^{2}+\mu_{I^{\prime }}^{2}+C_{1}\right)\left(\sigma_{I}^{2}+\sigma_{I^{\prime }}^{2}+C_{2}\right)}$$

where $C_{1}={\left(k_{1}\times L\right)}^{2}$, $C_{2}={\left(k_{2}\times L\right)}^{2}$, $k_{1}=0.01$, $k_{2}=0.03$, L=255. The $\mu_{I}$ and $\mu_{I^{\prime }}$ are the average values of the decrypted image $I^{\prime }$ and the original image *I*. The $\sigma_{I}$ and $\sigma_{I^{\prime }}$ are the variance values, and $\sigma_{II^{\prime }}$ is the covariance value between *I* and $I^{\prime }$. In this test, we also first encrypt 256×256 and 512×512 plain images at different CRs, and then decrypt these cipher images to obtain recovery images. Finally, we calculate the SSIM value between recovery images and plain images. Table 2 and Figure 5 show the SSIM result. The SSIM values are also increasing with CRs and the minimum value is also over 0.7, according to the results. It means that, in this indicator, our scheme also has very good compression recovery performance.

### 4.2. Key Space Analysis

The image cryptosystem requires enough key space to resist the brute-force attack. In our proposed image cryptosystem, the two 2D-SLIM and two 2D-LSCM hyper chaotic systems are used for controlling the permutation and diffusion process and for generating a measure matrix of compressive sensing. Hence, the real keys are two system parameters a01∈(0,3],a02∈(0,3] and four initial values x11∈(0,1),y11∈(0,1),x12∈(0,1),y12∈(0,1) of two 2D-SLIM systems, and two system parameters θ1∈(0,0.33)⋃(0.66,1),θ2∈(0,0.33)⋃(0.66,1) and four initial values xx0∈(0,1),yy0∈(0,1),xx1∈(0,1),yy1∈(0,1) of two 2D-LSCM systems. The change step of each initial value and parameters are $10^{-15}$, the key space can be calculated as $S={\left(3\times 10^{15}\right)}^{2}\times 0.66^{2}\times {\left(10^{15}\right)}^{10}=3.9204\times 10^{180}\approx 2^{600}$. Usually, if the key space is more than $2^{100}$, then we can consider that the image cryptosystem is good at resisting the brute-force attack [34].

### 4.3. Differential Attack

Differential attack is a method for analyzing keys from two cipher images that are encrypted by two tiny different plain images. The plain image sensitivity is an important feature for an image cryptosystem to resist differential attack. There are two measurements for evaluating the plain image sensitivity: the number of pixels change rate (NPCR) and unified average changing intensity (UACI) [12,13]. The NPCR and UACI are given by Equations (10) and (12), respectively.

(10) $$\mathrm{NPCR}=\frac{\sum_{i=1}^{M}\sum_{j=1}^{N}D\left(i,j\right)}{M\times N}\times 100\%,$$

where

(11) $$D\left(i,j\right)=\left\{\begin{array}{cc} 0, & C_{1}\left(i,j\right)=C_{2}\left(i,j\right)\\ 1, & C_{1}\left(i,j\right)\neq C_{2}\left(i,j\right) \end{array}\right.,$$

(12) $$\mathrm{UACI}=\frac{1}{M\times N}\left(\sum_{i=1}^{M}\sum_{j=1}^{N}\frac{\left|C_{1}\left(i,j\right)-C_{2}\left(i,j\right)\right|}{255}\right)\times 100\%,$$

where $C_{1}\left(i,j\right)$ and $C_{2}\left(i,j\right)$ are denoted as two cipher images that are generated by encrypting one-pixel different two plain images. *M* and *N* are the height and width of images, respectively. In order to evaluate the NPCR and UACI results, the critical values are given by Wu et al. [35]. The critical value of NPCR is given by:

(13) $$N_{\alpha }^{*}=\frac{Q-\Phi^{-1}\left(\alpha \right)\sqrt{Q/H}}{Q+1},$$

where *H* represents the total pixel numbers of image, *Q* represents the largest value that the pixels allowed in the image, and α is the significance level. When the test NPCR value is larger than critical value $N_{\alpha }^{*}$, we can consider that the proposed system has good plain image sensitivity.

The UACI critical interval $\left(U_{\alpha }^{*-},U_{\alpha }^{*+}\right)$ is given by:

(14) $$\left\{\begin{array}{c} u_{\alpha }^{*-}=\mu_{u}-\Phi^{-1}\left(\frac{\alpha }{2}\right)\sigma_{u},\\ u_{\alpha }^{*+}=\mu_{u}+\Phi^{-1}\left(\frac{\alpha }{2}\right)\sigma_{u}, \end{array}\right.$$

where

(15) $$\mu_{u}=\frac{Q+2}{3Q+3},$$

and

(16) $$\sigma_{u}=\sqrt{\frac{\left(Q+2\right)\left(Q^{2}+2Q+3\right)}{18{\left(Q+1\right)}^{2}QH}}.$$

If the UACI value falls into interval $\left(U_{\alpha }^{*-},U_{\alpha }^{*+}\right)$, then we can consider the two test images have enough difference. We assume significance level α=0.05. When the test image is 512×512, the NPCR critical value is $N_{0.05}^{*}=99.5893\%$ and the UACI critical interval is $\left(U_{0.05}^{*-},\;U_{0.05}^{*+}\right)=\left(33.3730\%,\;33.5541\%\right)$. When the test image is 256×256, the NPCR critical value is $N_{0.05}^{*}=99.5693\%$ and the UACI critical interval is $\left(U_{0.05}^{*-},\;U_{0.05}^{*+}\right)=\left(33.2824\%,\;33.6447\%\right)$.

**Figure 6:**
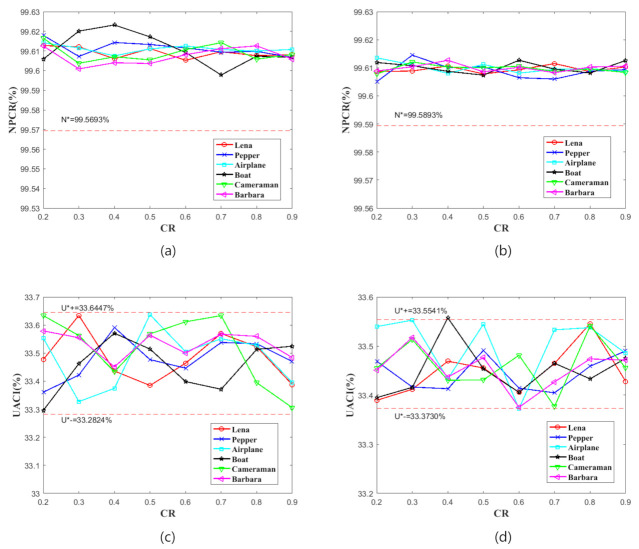
The number of pixels change rate (NPCR) and unified average changing intensity (UACI) test result. (**a**,**c**) show the results of images with the size of 256×256. (**b**,**d**) show the results of images with the size of 512×512. (**a**,**b**) show the results of NPCR tests. (**c**,**d**) show the results of UACI tests.

In this test, we complete the test 100 times for each CRs and calculate the average value, respectively. Table 3 and Figure 6 show the tests results. According to the data and figures, the NPCR and UACI test values are floating with CR changes. Nonetheless, all of the test values are basically within the critical values. The test result has shown that our proposed scheme is plain image enough in resisting the differential attack.

### 4.4. Statistical Analysis

#### 4.4.1. Histogram Analysis

A histogram can reflect the statistical feature of cipher image; the histogram is closer to uniform the better security performance. The histogram is shown in Figure 3. In Figure 3 rows (a, e, i), there are some cipher images that are encrypted in deferent CRs, and the corresponding histograms are shown in Figure 3 rows (b, f, j). The corresponding decrypted images are shown in Figure 3 rows (c, g, k). Figure 3 rows (d, h, l) shows the histograms of recovery image. The chi-squared test is used to evaluate the uniformity of cipher image’s histogram. Table 4 provides the chi-squared test results of cipher images, when the significance level is α=0.05. According to the results, our scheme has enough good diffused property to resist the statistical attack.

#### 4.4.2. Correlation Coefficient

As we all know, encryption is a process breaking the correlation of adjacent pixels. Therefore, correlation coefficient analysis is an important measurement for evaluating the permutation performance of image cryptosystem. The less correlation in cipher image, the better permutation performance.

The correlation coefficient can be calculated by Equation (17).

(17) $$r_{ab}=\frac{\mathrm{cov}(a,b)}{\sqrt{D(a)D(b)}}\;,$$

where *a* and *b* are two adjacent pixels’ gray values, and

(18) $$E\left(a\right)=\frac{1}{N}\sum_{i=1}^{N}a_{i},$$

(19) $$D\left(a\right)=\frac{1}{N}\sum_{i=1}^{N}{\left(a_{i}-E\left(a\right)\right)}^{2},$$

(20) $$\mathrm{cov}\left(a,b\right)=\frac{1}{N}\sum_{i=1}^{N}\left(a_{i}-E\left(a\right)\right)\left(b_{i}-E\left(b\right)\right).$$

**Figure 7:**
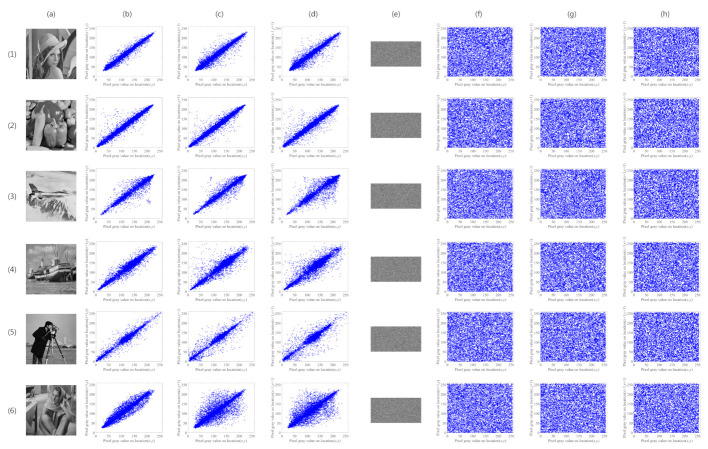
Correlation distributions. Rows (**1**–**6**) correspond to images of ’Lena’, ’Pepper’, ’Airplane’, ’Boat’, ’Cameraman’, and ’Barbara’, respectively; Column (**a**) shows the corresponding plain images; Columns (**b**–**d**) correspond to the plain images’ distributions of ’horizontal direction’, ‘vertical direction’, and ‘diagonal direction’, respectively; Column (**e**) is the corresponding cipher images by encryption; Columns (**f**–**h**) correspond to the cipher images’ distributions of ’horizontal direction’, ’vertical direction’, and ’diagonal direction’, respectively.

In this test, we first randomly select 10,000 pairs of adjacent pixels in the test image, and then calculate the correlation coefficient among these pixels. The test plain images are 512×512 and the cipher images are encrypted on CR=0.5. Table 5 shows the test results. The correlation distributions are shown in Figure 7 and the rows (**1**–**6**) correspond to images of ’Lena’, ’Pepper’, ’Airplane’, ’Boat’, ’Cameraman’, and ’Barbara’, respectively; Column (**a**) shows the corresponding plain images; Columns (**b**–**d**) correspond to the plain images’ distributions of ’horizontal direction’, ’vertical direction’, and ’diagonal direction’, respectively; Column (**e**) is the corresponding cipher images by encryption; Columns (**f**–**h**) correspond to the cipher images’ distributions of ’horizontal direction’, ’vertical direction’, and ’diagonal direction’, respectively.

### 4.5. Key Sensitivity Analysis

**Figure 8:**
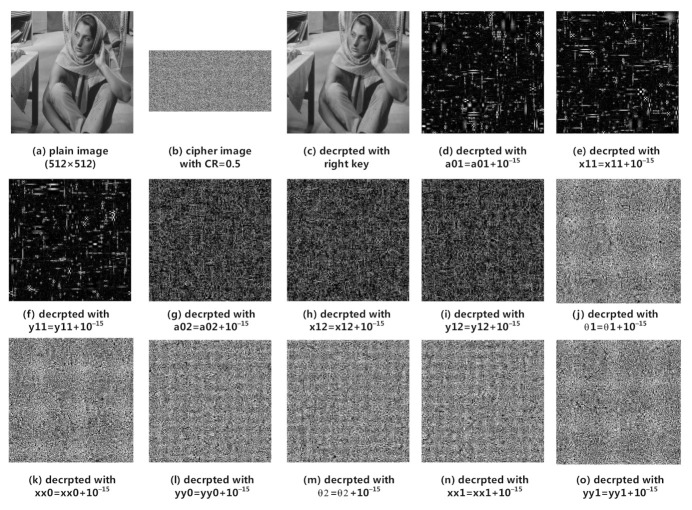
Decryption with tiny modified keys.

**Figure 9:**
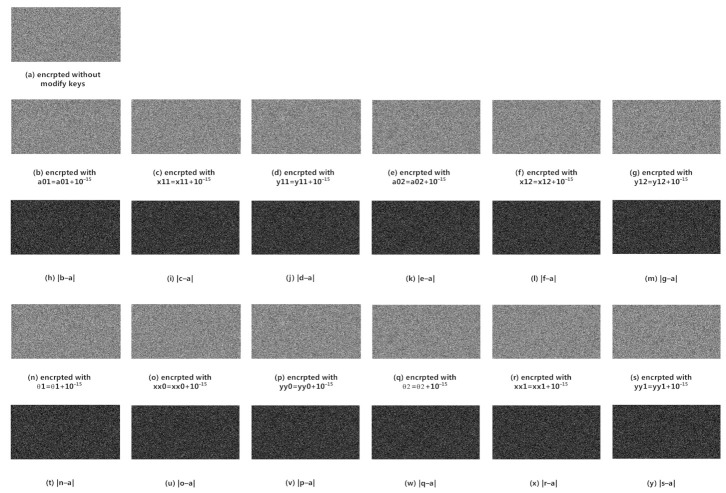
Comparison from encrypted images through tiny modified keys.

In this subsection, we will test the key sensitivity of our proposed image cryptosystem. In our scheme, there are 12 real keys, which are parameters and initial values of 2D-SLIM and 2D-LSCM systems. For this test, we select ’Barbara’ 512×512 as the test image. There are two tests for key sensitivity analysis. The first test we encrypted plain image on CR=0.5, and then decrypted with tiny modified keys. Figure 8 shows the test results. The second test is that we encrypt plain images with tiny modified keys and then compare the corresponding cipher images with without modified cipher images. Figure 9 shows the test results.

We quantitatively measure the difference between cipher images using NPCR and UACI. Table 6 shows the result. As the results of tests, our proposed scheme is very sensitive to the real keys.

### 4.6. Information Entropy

Global Shannon entropy (GSE) is used to evaluate the randomness of the whole image. The GSE is given by

(21) $$H\left(s\right)=\sum_{i=0}^{2^{K}-1}P\left(s_{i}\right)\log_{2}\frac{1}{P\left(s_{i}\right)},$$

where *K* is the gray level of the test image and $P(s_{i})$ means the probability of $s_{i}$. The GSE of 8-bit gray image is 8 bits in the ideal case. Table 7 shows the GSE results in different CRs.

**Figure 10:**
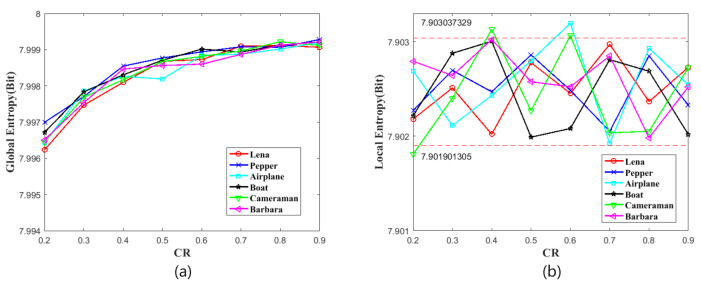
The information entropy analysis. (**a**) is global entropy. (**b**) is local entropy.

In order to further measure the randomness of cipher image, Wu et al. [36] introduced a method of local Shannon entropy (LSE). To calculate LSE, *k* non-overlapping image blocks $B_{1},B_{2},\cdots ,B_{k}$ with $T_{B}$ pixels are randomly selected from image *I*, and then the LSE is defined by:

(22) $$\bar{H_{k,T_{B}}}\left(I\right)=\sum_{i=1}^{k}\frac{H\left(B_{i}\right)}{k},$$

where $H(B_{i})$ is the GSE of image block $B_{i}$. For this test, the parameters $\left(k,T_{B}\right)=\left(30,\;1936\right)$ are selected. In this situation, the ideal value of LSE is 7.902469317. When the significance α=0.05, the tests passed when the test results fell into the interval (7.901901305,7.903037329). Table 8 shows the LSE test results. Figure 10 shows the global and local entropy analysis. According to this figure, the global entropies are increasing with CRs and the local entropies are floating with CRs. Nevertheless, the minimum of global entropy of 7.9962 entails sufficient security and the local entropies basically fall in security interval. Therefore, the information entropy results show that the cipher images that are generated by our proposed image cryptosystem have excellent randomness.

### 4.7. Robust Analysis

**Figure 11:**
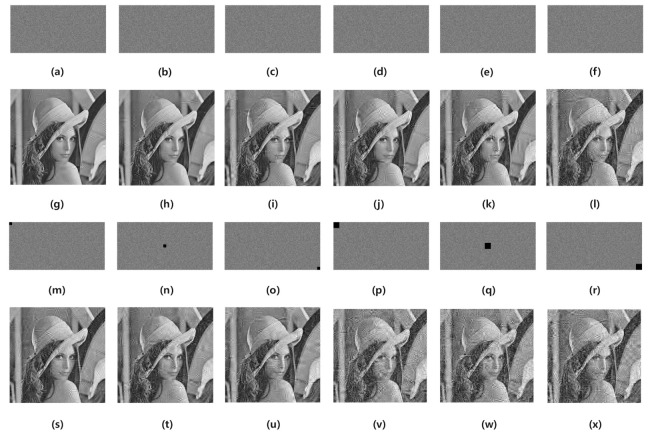
The robustness analysis result. (**a**–**f**) are cipher images with $1\times 10^{-4}$, $2\times 10^{-4}$, $3\times 10^{-4}$, $4\times 10^{-4}$, $5\times 10^{-4}$, $1\times 10^{-3}$ salt & pepper noise, respectively. (**g**–**l**) are corresponding decrypted images. (**m**–**o**) are the cipher images with 16×16 data lost. (**s**–**u**) are corresponding decrypted images. (**p**–**r**) are the cipher images with 32×32 data lost. (**v**–**x**) are corresponding decrypted images.

The robustness of the image cryptosystem means that some useful information can still be recovered when the cipher image is disturbed by noise or part of the data is lost during transmission. The robustness of the image cryptosystem in real communication applications is very important. In the test, some noise and different data loss amounts are added to Lena cipher images that are encrypted on CR =0.5 to evaluate the robustness of our proposed image cryptosystem. Figure 11 shows the test results. Most of the information in the plain image can still be identified from the decrypted image, as shown in Figure 11. Figure 11a–f are cipher images with $1\times 10^{-4}$, $2\times 10^{-4}$, $3\times 10^{-4}$, $4\times 10^{-4}$, $5\times 10^{-4}$, $1\times 10^{-3}$ salt & pepper noise, respectively. Figure 11g–l are corresponding decrypted images. Figure 11m–o are the cipher images with 16×16 data lost. Figure 11s–u are corresponding decrypted images. Figure 11p–r are the cipher images with 32×32 data lost. Figure 11v–x are corresponding decrypted images. It is shown that the algorithm has good robustness and it can be applied to practical scenarios.

### 4.8. Time Complexity Analysis

In order to evaluate the efficiency of our proposed image cryptosystem, we give the time complexity analysis and the time consuming of the simulation in this subsection. In this paper, we use two 2D-SLIM and two 2D-LSCM hyper chaotic systems to control the processes of compressive sensing and encryption, and it needs a total of Θ(3MN+N) iterations of computing floating point number. As we all know, there are many factors that affect the results of the actual test, such as hardware and software environments, programming languages, code optimization, parallel processing, programming skills, etc. Therefore, we give our simulation results of the time consumption under the environments that are mentioned at beginning of Section 4 and while using parallel computing technology. In our test, we encrypt and decrypt the same image 100 times, taking the average time. The encryption time of 256×256 Lena is 0.082 s and the decryption with CR =0.25, 0.5, 0.75 are 0.82 s, 1.23 s and 2.32 s, respectively. The encryption time of 512×512 Pepper is 0.336 s and the decryptions with CR =0.25, 0.5, and 0.75 are 3.23 s, 7.42 s, and 13.36 s, respectively.

### 4.9. Comparison with Other Works

In this section, we compare our proposed image cryptosystem with other recent works. For this comparison, we encrypt 512×512 8-bit gray level plain images ’Lena’ with CR =0.5. Table 9 shows the comparison result. The cipher image that is generated in Ref. [21] has poor randomness, because the global entropy is too low. Moreover, it also missing local entropy and plaintext sensitivity assessment. Ref. [22] presents the small key space and it is missing the information entropy assessment. Ref. [23] also has a small key space. Ref. [24] is missing the key space and a local entropy assessment. Our proposed image cryptosystem has the advantage of more comprehensive security performance, according to the comparison results.

## 5. Conclusions

In this paper, an efficient and secure plaintext-related chaotic image encryption scheme that is based on compressive sensing was proposed, which can simultaneously achieve the compression and encryption. In the proposed scheme, we generate the plaintext-sensitive internal keys to control the whole process of compression and encryption, which can make all processes have enough sensitivity to the plain image. The permutation that was controlled by the two-dimensional Sine improved Logistic iterative chaotic map (2D-SLIM) has been applied to the coefficient matrix in order to make the energy of matrix dispersive. A plaintext related compressive sensing was used to reduce the data storage capacity while the privacy of image is guaranteed. Additionally, we make sure the cipher image lower correlation and distribute uniform by quantifying the measurement results to 0∼255 and doing permutation and diffusion under the controlling by two-dimensional Logistic-Sine-coupling map (2D-LSCM). Finally, some common compression and security performance analysis methods are used for testing our scheme. The tests and comparison results have shown that our proposed scheme has both excellent security and compression performance in order to ensure the digital image application in the network. The image encryption combining compressive sensing is still under constant research, and there are still many problems that need to be further studied and solved. In the next stage, we will focus on the multi-image aggregation encryption and parallel block compressed sensing.

## Figures and Tables

**Table 1:** The peak signal to noise ratio (PSNR) test results.

Images	CR = 0.2	CR = 0.3	CR = 0.4	CR = 0.5	CR = 0.6	CR = 0.7	CR = 0.8	CR = 0.9
Lena (256×256)	32.8060	34.3257	35.5494	36.2231	37.0150	37.7089	38.4807	39.1588
Lena (512×512)	35.4609	36.6669	37.5536	38.4038	38.8188	39.3051	39.6735	40.0186
Pepper (256×256)	32.3384	33.9538	35.2645	36.3140	37.2489	37.9024	38.5522	39.4550
Pepper (512×512)	35.1376	36.1261	36.8545	37.1843	37.5528	37.9703	38.1828	38.2197
Airplane (256×256)	32.1119	33.1697	34.0963	34.7593	35.2559	35.6004	36.3997	36.9712
Airplane (512×512)	34.1957	35.0839	35.6081	36.2459	37.0352	37.2760	37.4211	37.7294
Boat (256×256)	31.7675	32.5097	33.3848	33.9783	34.5981	35.2112	35.7384	36.2905
Boat (512×512)	32.8568	33.9273	34.4103	35.1400	35.7692	36.4291	36.7441	37.3474
Cameraman (256×256)	31.9799	33.1808	33.8290	34.4296	35.0475	35.5772	36.1571	36.7097
Cameraman (512×512)	36.0659	37.7784	39.2547	39.8190	40.6606	40.7227	40.7004	41.4673
Barbara (256×256)	32.3834	33.3900	34.2665	35.1264	35.7329	36.4359	36.9932	37.6480
Barbara (512×512)	32.3296	33.3472	34.1002	34.9419	35.8160	36.5540	37.3483	37.7838

**Table 2:** The Structural Similarity Index Measurement (SSIM) test results.

Images	CR = 0.2	CR = 0.3	CR = 0.4	CR = 0.5	CR = 0.6	CR = 0.7	CR = 0.8	CR = 0.9
Lena (256×256)	0.7740	0.8476	0.8859	0.9018	0.9150	0.9262	0.9357	0.9424
Lena (512×512)	0.8278	0.8549	0.8713	0.8862	0.8909	0.8983	0.9034	0.9081
Pepper (256×256)	0.7603	0.8338	0.8716	0.8947	0.9097	0.9165	0.9227	0.9337
Pepper (512×512)	0.7911	0.8141	0.8309	0.8368	0.8458	0.8568	0.8616	0.8631
Airplane (256×256)	0.7303	0.8018	0.8429	0.8643	0.8713	0.8705	0.8901	0.8988
Airplane (512×512)	0.7988	0.8190	0.8254	0.8405	0.8586	0.8587	0.8581	0.8615
Boat (256×256)	0.7015	0.7670	0.8184	0.8425	0.8640	0.8816	0.8946	0.9050
Boat (512×512)	0.7330	0.7860	0.8012	0.8247	0.8424	0.8587	0.8656	0.8793
Cameraman (256×256)	0.6885	0.7740	0.8086	0.8332	0.8525	0.8664	0.8780	0.8873
Cameraman (512×512)	0.8567	0.8850	0.9076	0.9104	0.9202	0.9184	0.9149	0.9261
Barbara (256×256)	0.7509	0.8061	0.8424	0.8706	0.8864	0.9034	0.9136	0.9250
Barbara (512×512)	0.7644	0.8214	0.8471	0.8693	0.8886	0.8992	0.9122	0.9142

**Table 3:** Number of pixels change rate (NPCR) and unified average changing intensity (UACI) results.

Image	CR = 0.2		CR = 0.5		CR = 0.8	
	NPCR(%)	UACI(%)	NPCR(%)	UACI(%)	NPCR(%)	UACI(%)
Lena (256×256)	99.6127	33.4770	99.6112	33.3843	99.6076	33.5224
Lena (512×512)	99.6086	33.3894	99.6078	33.4552	99.6086	33.5457
Pepper (256×256)	99.6179	33.3606	99.6132	33.4766	99.6097	33.5329
Pepper (512×512)	99.6050	33.4691	99.6103	33.4910	99.6087	33.4590
Airplane (256×256)	99.6142	33.5532	99.6110	33.6366	99.6099	33.5275
Airplane (512×512)	99.6135	33.5396	99.6112	33.5445	99.6097	33.5375
Boat (256×256)	99.6058	33.2956	99.6171	33.5142	99.6073	33.5132
Boat (512×512)	99.6119	33.3949	99.6073	33.4537	99.6081	33.4332
Cameraman (256×256)	99.6164	33.6333	99.6054	33.5684	99.6058	33.3942
Cameraman (512×512)	99.6078	33.4550	99.6100	33.4309	99.6095	33.5411
Barbara (256×256)	99.6125	33.5789	99.6036	33.5636	99.6125	33.5594
Barbara (512×512)	99.6088	33.4506	99.6085	33.4766	99.6102	33.4737

**Table 4:** Histogram uniformity evaluation by chi-squared test (*p*-value).

Images	CR = 0.2	CR = 0.3	CR = 0.4	CR = 0.5	CR = 0.6	CR = 0.7	CR = 0.8	CR = 0.9
Lena (256×256)	0.7183	0.9713	0.7252	0.3415	0.4128	0.8003	0.8271	0.7778
Lena (512×512)	0.2174	0.1773	0.1874	0.7268	0.1592	0.8726	0.4672	0.1685
Pepper (256×256)	0.3111	0.2117	0.2766	0.2717	0.3582	0.9891	0.6976	0.7083
Pepper (512×512)	0.6795	0.8444	0.9260	0.9765	0.1287	0.4755	0.9823	0.5199
Airplane (256×256)	0.7867	0.1359	0.5739	0.3754	0.1576	0.9868	0.3256	0.9142
Airplane (512×512)	0.9632	0.5859	0.9817	0.9213	0.8393	0.8304	0.2412	0.8186
Boat (256×256)	0.9429	0.4776	0.4672	0.1594	0.4086	0.7931	0.9114	0.7712
Boat (512×512)	0.5941	0.8562	0.3941	0.8378	0.8965	0.2870	0.7980	0.9894
Cameraman (256×256)	0.5087	0.7340	0.7240	0.2415	0.2752	0.2131	0.3656	0.3492
Cameraman (512×512)	0.4639	0.5619	0.3150	0.7148	0.4347	0.3540	0.8971	0.1729
Barbara (256×256)	0.4548	0.5059	0.2825	0.2531	0.3462	0.2301	0.1449	0.3510
Barbara (512×512)	0.5269	0.2898	0.9238	0.3938	0.3166	0.2771	0.5193	0.4375

**Table 5:** Correlation coefficients.

Image	Horizontal		Vertical		Diagonal	
	Plain	Cipher	Plain	Cipher	Plain	Cipher
Lena (512×512)	0.9852	0.00057	0.9714	0.0028	0.9587	−0.0014
Pepper (512×512)	0.9801	0.0017	0.9778	0.0013	0.9643	0.00077
Airplane (512×512)	0.9653	0.0046	0.9678	0.0065	0.9369	0.0026
Boat (512×512)	0.9723	0.0012	0.9403	−0.0091	0.9238	0.0071
Cameraman (512×512)	0.9898	0.0019	0.9833	0.0043	0.9711	0.0056
Barbara (512×512)	0.9596	−0.0039	0.8610	0.0022	0.8406	0.00067

**Table 6:** Quantitative analysis of key sensitivity.

Cipher Image with Modify Key	NPCR (%)	UACI (%)
$a01=a01+10^{-15}$	97.4136	30.0742
$x11=x11+10^{-15}$	98.8632	33.8622
$y11=y11+10^{-15}$	97.9836	32.8013
$a02=a02+10^{-15}$	96.9574	31.1781
$x12=x12+10^{-15}$	97.0551	31.6505
$y12=y12+10^{-15}$	98.6200	32.6244
$\theta 1=\theta 1+10^{-15}$	99.6124	33.5163
$xx0=xx0+10^{-15}$	99.5987	33.4155
$yy0=yy0+10^{-15}$	99.5819	33.5568
$\theta 2=\theta 2+10^{-15}$	99.5796	33.5179
$xx1=xx1+10^{-15}$	99.6002	33.4799
$yy1=yy1+10^{-15}$	99.6185	33.5206

**Table 7:** Global Information entropy.

Images	CR = 0.2	CR = 0.3	CR = 0.4	CR = 0.5	CR = 0.6	CR = 0.7	CR = 0.8	CR = 0.9
Lena (512×512)	7.9962	7.9974	7.9980	7.9986	7.9987	7.9990	7.9991	7.9990
Pepper (512×512)	7.9969	7.9977	7.9985	7.9987	7.9989	7.9990	7.9990	7.9992
Airplane (512×512)	7.9964	7.9978	7.9982	7.9981	7.9988	7.9988	7.9990	7.9992
Boat (512×512)	7.9967	7.9978	7.9983	7.9987	7.9990	7.9989	7.9991	7.9992
Cameraman (512×512)	7.9964	7.9976	7.9981	7.9986	7.9988	7.9989	7.9992	7.9991
Barbara (512×512)	7.9965	7.9975	7.9984	7.9985	7.9985	7.9988	7.9991	7.9992

**Table 8:** The local Shannon entropy test.

Images	CR = 0.2	CR = 0.3	CR = 0.4	CR = 0.5	CR = 0.6	CR = 0.7	CR = 0.8	CR = 0.9
Lena (512×512)	7.9022	7.9025	7.9020	7.9028	7.9025	7.9030	7.9024	7.9027
Pepper (512×512)	7.9023	7.9027	7.9025	7.9029	7.9025	7.9021	7.9029	7.9023
Airplane (512×512)	7.9027	7.9021	7.9024	7.9028	7.9032	7.9019	7.9029	7.9025
Boat (512×512)	7.9022	7.9029	7.9030	7.9020	7.9021	7.9028	7.9027	7.9020
Cameraman (512×512)	7.9018	7.9024	7.9031	7.9023	7.9031	7.9020	7.9021	7.9027
Barbara (512×512)	7.9028	7.9026	7.9030	7.9026	7.9025	7.9028	7.9020	7.9025

**Table 9:** The comparison result.

Algorithms	Cipher Correlation Coefficients			Global	Local	Key Space	Plaintext Sensitivity		PSNR (dB)
	Horizontal	Vertical	Diagonal	Entropy	Entropy		NPCR (%)	UACI (%)	
Our work	0.00057	0.0028	−0.0014	7.9986	7.9028	$2^{600}$	99.6078	33.4552	38.3438
Ref. [21]	0.0061	0.0018	−0.0024	5.0508	-	$1.15\times 10^{105}$	-	-	-
Ref. [22]	−0.0016	−0.0010	−0.0015	-	-	$2^{149}$	99.6061	33.4150	35.51
Ref. [23]	0.0016	0.0081	−0.0016	7.9974	7.9027	$2^{176}$	99.6201	33.5247	30.8184
Ref. [24]	−0.0028	−0.0096	−0.0030	7.9960	-	-	99.60	34.17	39.04

## Data Availability

The data used to support the findings of this study are included within the article.

## Abbreviations

The following abbreviations are used in this manuscript:

2D-SLIM	two-dimensional Sine improved Logistic iterative chaotic map
2D-LSCM	two-dimensional Logistic-Sine-coupling map
DES	Data Encryption Standard
AES	Advanced Encryption Standard
DNA	deoxyribonucleic acid
CS	Compressed sensing
CR(s)	compression ratio(s)
RIP	restricted isometry property
RIC	Restricted Isometry Constant
DWT	Discrete Wavelet Transform
PSNR	Peak signal to noise ratio
SSIM	Structural similarity index measurement
NPCR	number of pixels change rate
UACI	unified average changing intensity
